# Looking beyond the neonatal: what does administrative data tell us about the preschool health and developmental outcomes of children exposed to opioids in utero?

**DOI:** 10.23889/ijpds.v9i5.2580

**Published:** 2024-09-18

**Authors:** Louise Marryat, Senga Robertson-Albertyn, James P Boardman, Alison McFadden, Anne Whittaker

**Affiliations:** 1 University of Dundee; 2 University of Edinburgh; 3 University of Stirling

## Background

The world is facing an opioid epidemic. Children of women who use opioids in pregnancy are difficult to follow-up over long periods using traditional research methods due to the complexity of their lives. Resultingly, we have little robust evidence on their longer-term outcomes.

## Objective

This study aimed to explore the impact of illicit and prescription opioid exposure in pregnancy on preschool health and developmental outcomes.

## Approach

Data identified 6,408 children (born 2009-2019 in Scotland) exposed to opioids through illicit use and/or medication assisted treatment  (MAT), alongside a matched control group (n.19,089). Regression models will examine associations between opioid exposure and key outcomes up to age 5, including the Ages and Stages Questionnaire, accidents and injuries, and chronic health conditions such as asthma, controlling for other risk factors (e.g. alcohol use in pregnancy, gestation).

## Results

Early results indicated differences in neonatal outcomes, with poorer outcomes for the illicit opioid/MAT cohort in terms of being born early, having lower birthweight, length and head circumference, and being more likely to be removed from their mother prior hospital discharge. Modelling of preschool outcomes is underway and will be finished in June 2024.

## Conclusions

Having proven that we can identify these children in administrative data, this paper will present cutting-edge data on the impact of exposure to illicit and prescription opioids on preschool outcomes. This will provide robust evidence on these impacts, and highlight where additional support might be required for these children from birth to starting school.

